# Healthier side dishes at restaurants: an analysis of children’s perspectives, menu content, and energy impacts

**DOI:** 10.1186/1479-5868-11-81

**Published:** 2014-07-04

**Authors:** Stephanie Anzman-Frasca, Franciel Dawes, Sarah Sliwa, Peter R Dolan, Miriam E Nelson, Kyle Washburn, Christina D Economos

**Affiliations:** 1 Child-Obesity-180, Tufts University, 150 Harrison Avenue, Boston, MA 02111, USA; 2 Gerald J. and Dorothy R. Friedman School of Nutrition Science and Policy, Tufts University, 150 Harrison Avenue, Boston, MA 02111, USA; 3 Frances Stern Nutrition Center, Tufts Medical Center, 150 Harrison Avenue, Boston, MA 02111, USA

**Keywords:** Restaurants, Children, Food away from home, Fruit, Vegetables, Side dishes, Defaults, Healthy eating, Childhood obesity

## Abstract

**Background:**

Children consume restaurant-prepared foods at high rates, suggesting that interventions and policies targeting consumption of these foods have the potential to improve diet quality and attenuate excess energy intake. One approach to encouraging healthier dietary intake in restaurants is to offer fruits and vegetables (FV) as side dishes, as opposed to traditional, energy-dense accompaniments like French fries. The aims of the current study were to examine: children's views about healthier side dishes at restaurants; current side dish offerings on children's menus at leading restaurants; and potential energy reductions when substituting FV side dishes in place of French fries.

**Methods:**

To investigate children’s attitudes, a survey was administered to a nationally representative sample of U.S. 8- to 18-year-olds (n = 1178). To examine current side dish offerings, children's menus from leading quick service (QSR; n = 10) and full service restaurant chains (FSR; n = 10) were analyzed. Energy reductions that could result from substituting commonly-offered FV side dishes for French fries were estimated using nutrition information corresponding to the children's menu items.

**Results:**

Two-thirds of children reported that they would not feel negatively about receiving FV sides instead of French fries with kids' meals. Liking/taste was the most common reason that children gave to explain their attitudes about FV side dishes. Nearly all restaurants offered at least 1 FV side dish option, but at most restaurants (60% of QSR; 70% of FSR), FV sides were never served by default. Substituting FV side dishes for French fries yielded an average estimated energy reduction of at least 170 calories.

**Conclusions:**

Results highlight some healthy trends in the restaurant context, including the majority of children reporting non-negative attitudes about FV side dishes and the consistent availability of FV side dish options at leading QSR and FSR. Yet the minority of restaurants offer these FV sides by default. Promoting creative, appealing FV side dishes can result in healthier, less energy-dense meals for children. Substituting or displacing energy-dense default side dishes with such FV dishes show promise as part of continued, comprehensive efforts to increase the healthfulness of meals consumed by children in restaurant settings.

## Background

Over the past few decades, U.S. children's eating patterns have shifted, including an increased contribution of foods prepared away from home to children's overall dietary intake [1]. National surveillance data show that 6-to-11-year-old children consumed 11% of daily energy from quick service restaurants (QSR) and 5% from full service restaurants (FSR) in 2007–2008; for adolescents aged 12–19, these proportions were 17% and 7% respectively [2]. Restaurant-prepared foods tend to be higher in energy and fat and lower in nutritional quality compared to foods prepared at home [3], and accordingly, consumption of foods from QSR and FSR has been associated with higher energy intake and lower diet quality in nationally representative samples of children [4]. Given American children’s frequent consumption of food from restaurants, policies and interventions addressing dietary intake in these settings have the potential to improve children’s diet quality and energy balance.

Some restaurants are engaged in efforts to improve the nutritional quality of their children's menu offerings [5], but concerns about energy consumed in restaurant settings persist. Evaluations of children’s menus at select restaurants have revealed the ubiquity of French fries as a side dish [6] and low overall diet quality scores, driven in part by low availability of dark green and orange vegetables, legumes, and whole grains [7]. Further, a report released in 2013 showed that 91% of children’s meals at the top 50 restaurant chains did not meet the National Restaurant Association’s Kids LiveWell nutrition standards [8], and with regards to energy content specifically, 50% of children's meals did not meet the Kids LiveWell criterion of 600 calories or less. These findings justify efforts to decrease the energy content of children's meals, as the appropriate energy content of a meal for a sedentary child ranges from 400–667 calories, depending on their age and sex [9].

Several recent policy approaches are congruent with the goal of promoting healthier eating in restaurants, including regulations focused on menu labeling and marketing. Both of these approaches address consumers’ access to information about food, and thus far, there are mixed results in terms of the potential impacts of these strategies on eating behavior [10-23]. While evaluations of these regulatory initiatives are ongoing, a third potential way to decrease energy consumed in restaurant settings is supported by evidence from other domains. This potential shift involves making the healthier choice the easy choice, by making it more explicit or prominent, or by making it the default option [24]. Behavioral economics research has demonstrated that individuals are more likely to accept the default option for organ donation, savings plans, car insurance, and email subscriptions [25-28]. For example, when automobile insurance customers were required to opt in to obtain additional coverage, 20% of customers participated, while requiring them to opt out resulted in 75% participation in the same additional coverage [28]. In the restaurant industry, it is common to offer default side dish items, without asking customers if another item would be preferred [29]. Traditionally, the default accompaniments on restaurant menus are energy dense and nutrient poor, such as French fries and sugar-sweetened beverages [10]. It follows that energy consumed in restaurants may be reduced by decreasing the automaticity of such items and by increasing the accessibility of healthier, less energy-dense side items, like fruits and vegetables (FV). Specific strategies could include: 1) adding more healthy items to the possible side dish options offered on children's menus, 2) replacing energy-dense side dishes with healthier default side dishes, or 3) displacing energy-dense side dishes by reducing their portion size and adding a serving of a healthier food by default.

Little research has examined these specific strategies. In one study, the purchasing of energy-dense default side dishes decreased after adding healthier side dish options to children’s menus, suggesting that adding healthier side dish options may positively impact dietary intake [30]. Additionally, recent research revealed that the energy content of children’s meal orders decreased following the implementation of a displacement strategy, in which the portion size of French fries was decreased and a serving of apples was included with all children’s meals by default [31]. These two studies, each within an individual QSR chain, provide initial evidence that increasing the accessibility and automaticity of healthier side dishes could have positive impacts on children’s dietary intake in restaurant settings. Less is known about children’s perceptions about the addition or substitution of healthier side dish options.

Traditionally, research has indicated that children and adolescents select and consume foods based on taste, appearance, and familiarity [32,33]. To facilitate conversations about feasible, large-scale menu modifications, more information is needed regarding children’s attitudes about having healthier side dishes, such as non-fried FV, as default side dishes that replace or displace traditional choices like French fries in restaurant settings. Children's current perceptions about this approach would be of interest to restaurants with concerns about the feasibility of healthy menu modifications, as well as researchers, caregivers, and policymakers. Thus, the first aim of this study was to assess children’s views about eating at restaurants, including attitudes about FV side dishes, in a nationally representative sample of 8- to 18-year-olds. Secondly, to explore whether children’s perspectives aligned with restaurant offerings, we analyzed current side dish offerings using children’s menus and corresponding nutrition information from 20 leading QSR and FSR. Finally, we estimated the energy reductions that would result from substituting the most common FV side dishes for French fries.

## Methods

The methodology of this study is split into two sections. The first section is focused on the assessment of children's perceptions. The second section is focused on the analysis of children's menus and corresponding nutrition information from 20 leading QSR and FSR.

### Part I: assessing children’s perceptions about restaurants

#### ***Participants***

Respondents were 8- to 18-year-old U.S. children (n = 1178). Sampling weights based on age, sex, race/ethnicity, parental education, urbanicity, and region were incorporated into analyses of survey responses, so that overall results would be representative of 8- to 18-year-old children in the U.S. All human subjects procedures were approved by the Tufts University Institutional Review Board.

#### ***Procedure***

Harris Interactive (New York) was commissioned by child obesity researchers at Tufts University to conduct an online survey focused on youth eating habits within the United States. Survey questions were fielded as part of a larger survey covering a range of topics, in September 2010. The current study focuses on nine questions about restaurants. The sample was obtained from the Harris Poll Online (HPOL) opt-in panel of millions of respondents. Invitations for the HPOL panel were emailed to a stratified random sample identified as U.S. residents ages 13–18 and to a stratified random sample identified as U.S. residents ages 18 years or older with a 8–17 year old child in the household. Respondents were invited to participate in the survey through password-protected email invitations. The HPOL panel was recruited through hundreds of sources using diverse recruitment methods in order to minimize selection bias.

Participants were asked how often they eat at restaurants and how often they eat food from restaurants as take-out (response options for each: never, a few times a year, a few times a month, 1–3 times a week, or 4 or more times a week). Participants who indicated ever eating food from restaurants were asked what they do when they are at a restaurant and there is too much food (take it home, leave it on plate, try to finish it anyway, give it to someone else, there is never too much food, other) and how often they order kids’ meals (never, rarely, sometimes, very often). Participants who indicated that they ever ate kids’ meals were then asked how likely they would be to order a kid’s meal that came with: 1) vegetables such as a salad, green beans, or carrots; 2) French fries; and 3) fruits such as apple slices, orange slices, or a fruit cup (response options for each of these three items: not at all likely, not too likely, somewhat likely, very likely). These participants were also asked how they would feel if their kid’s meal came with a vegetable or fruit but not French fries (very unhappy, somewhat unhappy, neither unhappy nor happy, somewhat happy, very happy). Finally, participants who indicated that they would be somewhat or very unhappy and participants who indicated that they would be somewhat or very happy were asked to provide an open-ended response explaining why. When examining internal consistency between the four ordinal outcome variables assessing the acceptability of different side dish options, the resulting Cronbach’s alpha value was .52, and after removing the item assessing the likelihood of ordering French fries, which demonstrated low variability, the alpha value increased to .74.

#### ***Data analysis***

All analyses of survey items and demographic data incorporated sampling weights, so that results would be representative of 8- to 18-year-olds in the U.S. Survey data were analyzed using SAS 9.2 (Cary, NC). Frequencies were calculated to determine children’s rates of eating at restaurants and eating take-out, as well as their behavior when served too much food at restaurants, rates of ordering kids’ meals, and attitudes about different side dish options, overall and by age group (tweens, age 8–12, and teens, age 13–18). Eight ordinary least squares regression models were conducted to test the frequency of eating at restaurants and the frequency of eating take-out each as predictors of 1) children’s reported likelihood of ordering a kid's meal that came with vegetables, 2) their reported likelihood of ordering a kid's meal that came with French fries, 3) their reported likelihood of ordering a kid's meal that came with fruit, and 4) their attitudes about receiving a vegetable or fruit instead of French fries in their kid's meal. Child age group (tweens vs. teens) and sex were included as covariates in all models.

Open-ended responses, in which children explained why they would be happy or unhappy about FV side dishes, were coded in multiple passes. First, two researchers read all of the open-ended responses and generated a coding scheme with categories that subsumed all of the responses (Table 1). Then, the children’s responses were coded independently by two other researchers, who selected all categories that applied for each open-ended response, entering dummy-coded data for each category into Microsoft Excel. The two researchers’ coded data were compared, and in instances where their responses did not agree (3% of cases), the appropriate response was determined and entered by a researcher who generated the coding scheme. Frequencies were calculated depicting the percent of children who endorsed each reason. We also conducted logistic regression models, in which children’s frequency of eating at restaurants, age group (tweens or teens), and sex were tested as predictors of the likelihood of endorsing the different reasons for being happy/unhappy. As with the quantitative items, sampling weights were incorporated in analyses of open-ended responses. These analyses do not include children who would feel neutral about FV side dishes, as they were not asked to provide an open-ended response; we also excluded a small number of open-ended responses (n = 8) where children’s responses directly contradicted their answer to the previous, closed-ended question about whether they would be happy or unhappy.

**Table 1:** Categories used to code children's open-ended responses about kids' meals coming with fruits or vegetables instead of French fries

**Categories**	**Sample responses**
***For children who would be happy about the substitution:***	
1. Liking/taste. Included the following:	
I like the taste of fruits and vegetables	“Fruits or vegetables taste just as good sometimes better”
I like the taste of fruits	“I like fruit in a meal for dessert after. Fruit tastes great.”
I like the taste of vegetables	“I love veggies and I also love love love salads of all kinds.”
I don’t like/don’t prefer French fries	“Because i really don't care for french fries too much anymore”
2. Health: Fruits and vegetables are healthy	“Because this is better for you”
3. Choice: I want choices/variety	“I like to eat all kinds of things. I can always get French Fries.”
4. Treat: Fruits and vegetables are a treat	“Because I rarely ever get fruits and vegetables and I love them.”
5. Other	“I am allergic to msg alots of fries have msg on them.”
6. Don’t know	“I don’t know,” “Because”
***For children who would be unhappy about the substitution:***	
1. Liking/taste. Included the following:	
I like the taste of French fries	“I like french fries more than any other side dish.”
I don’t like/don’t prefer fruit and don’t like/don’t prefer vegetables	“I don't like vegetables and fruit as much as fries”
I don’t like/don’t prefer fruit	“I do not eat a lot of fruit”
I don’t like/don’t prefer vegetables	“Vegetables don't taste good. I only like certain fruits.”
2. Habit: French fries are what I’m used to	“I always get french fries and im not used to have fruit with fast food”
3. Choice: I want to have a choice	“Well I guess it depends on what I'm eating because french fires taste better with certain dishes.”
4. Treat: French fries are a treat	“Because i like to eat fries when i go out i eat enough vegetiables and fruit at home”
5. Other	“I like to eat meet”
6. Don’t know	“Don’t know,” “Because”

Note: Spelling and grammar errors were retained to capture the children's voices accurately. Also note that coders selected all categories that applied to a given response.

### Part II: menu analysis

Data coded from children's menus and the corresponding nutrition information were entered into Microsoft Excel, and analyses of these data were conducted using SAS 9.2 and Stata 10.1 (College Station, TX). To place children's perceptions in the context of current restaurant offerings, we analyzed the availability of FV side dishes on the children's menus of the top 10 QSR and top 10 FSR chains (by sales; [34]) that met our inclusion criteria of: 1) having a children's menu, and 2) offering side dishes. These two restaurant segments were selected because they are the major segments represented among the top 25 restaurants overall (92%; [34]). In defining side dishes, we included all items that were explicitly listed on the menu as side dishes (e.g., all main dishes come with a side of apples; with each main dish, customers choose from a list of side dish options; n = 19 restaurants had at least some kids’ meals in this category). Additionally, for pre-determined, multi-component meals, we viewed each available main dish and considered anything listed explicitly as an accompaniment to the main dish to be a side dish (e.g., grilled cheese served with mixed fruit cup; pancakes served with fruit and bacon; n = 3 restaurants had at least some kids’ meals in this category). Garnishes were not considered to be individual side dishes; garnishes were defined as items that would not be expected to stand alone and that served primarily to modify the main or side dish (e.g., whipped cream served atop a pancake, dip served with celery). Such garnishes typically contribute less than a serving of the respective item and often serve a decorative, rather than substantive, purpose. Thus, these items were considered to be a part of the main or side dish that they modified, rather than an independent dish. We also excluded from our study any side dishes that involved an extra charge, as our intent was to examine the side dishes that were available as part of a children's meal in its standard form and price.

The children's menus from the 20 restaurants were accessed on restaurant websites in April 2013, and two researchers coded available side dishes on four dimensions: 1) whether a FV was a possible side dish item (yes/no), 2) the percent of available side dishes that were FV, 3) whether a FV was the automatic or default side dish (for all meals, for some meals, for no meals), and 4) which specific FV were available as side dishes. Default status was coded based on the way the meals were listed on the online menus. For example, if the menu indicated that all kids' meals were served with a side of apples, then a FV was the default side dish for all meals. If the menu indicated that the child was to choose a side from a list of options that included FV as well as non-FV sides, then a FV was the default side for no meals. Coders were provided with lists of vegetables and fruits, as defined by the United States Department of Agriculture [35]. Fried foods were not counted as FV. Legumes were considered vegetables, following the Dietary Guidelines for Americans and MyPlate [9,35]. Coders used these lists to determine whether available sides were FV and to indicate which specific FV were available as side dishes: they indicated whether each FV item was present (1) as a side dish option or not (0) on each menu; if there were two versions of the FV item available on a particular restaurant’s menu (e.g., corn on the cob and corn kernels), the corresponding item was coded accordingly (e.g., as a “2” instead of a “1”; this occurred in the case of 5 of 380 coded data points).

The two researchers’ coded menu data were compared, and in instances where their responses did not agree (5%), the appropriate response was determined and entered by a researcher who generated the coding scheme. Frequencies were calculated to depict the percent of QSR and FSR offering FV as possible side dishes and as default side dishes. The percent of side dishes that were FV was averaged across restaurants, overall and by segment. Frequencies were calculated to depict the prevalence of each FV side item across the children’s menus (e.g., apples, broccoli, non-fried potatoes), and these were also rank ordered to capture the most commonly offered FV side items, overall and by segment. The list of restaurants and more detail about the menu coding scheme are provided in Table 2. Lastly, nutrition information was collected from restaurant websites when available (n = 19 of 20 restaurants) and was used to determine total energy content of all non-fried FV side dishes offered on children’s menus, as well as for the most common non-FV side dish, which was French fries (offered at 13 of 20 restaurants). To demonstrate the potential energy reduction attained by substituting a non-fried FV side dish for French fries, the sample for this final aim was restricted to restaurants offering both French fries (including curly fries, waffle fries, and tater tots) and at least one of the five FV side dishes most commonly offered with, or instead of, French fries (apple slices, apple sauce, mashed potatoes, broccoli, or salad; n = 11 restaurants). For each restaurant, the potential energy reduction was calculated by subtracting the energy content of each applicable FV side dish from the energy content of that restaurant's serving of French fries. These values were averaged across restaurants separately for each FV item. Energy from dips or dressings were included when these were served alongside FV sides if this information was available.

**Table 2:** Coding scheme for children’s menus at top 10 quick service and top 10 full service restaurants

**Variables coded from each menu**	**Description**
1. Availability of a FV side dish	Whether there was at least one FV side available on the children’s menu (1) or not (0)
2. Prevalence of FV side dishes	The number of FV side dishes divided by the total number of side dishes on each children’s menu
3. Default status of FV side dishes	Restaurants were coded based on the default side dish options on their children’s menu:
1 = FV always the default	Included restaurants in which a FV was always the default side dish and restaurants in which there was no default, but all side dishes offered were FV
2 = FV sometimes the default	Included restaurants in which the side dish depended on the main dish ordered: some meals came with a FV side dish by default while others did not
3 = FV never the default	Included restaurants in which a non-FV was always the default side dish and restaurants in which there were no default side dishes (and possible side dish options included non-FV)
4. Types of FV side dishes:	Whether each of the FV side dish items listed below (n = 19 possibilities across restaurants) were included on the children’s menu (0 = no, 1 = yes, 2 = two distinct forms of that item were included as side dish options; e.g., corn on the cob and corn kernels).
• Apples	
• Applesauce	
• Mixed fruit	
• Grapes	
• Oranges	
• Non-fried potatoes	
• Corn	
• Green beans	
• Broccoli	
• Carrots	
• Celery	
• Salad	
• Mixed vegetables	
• Pineapple	
• Baked beans	
• Cole slaw	
• Berries	
• Other beans	
• Greens	

Note: The top 10 quick service restaurants were: McDonald’s, Subway, Burger King, Wendy’s, Taco Bell, KFC, Chik-Fil-A, Sonic, Jack-in-the-box, Arby’s. The top 10 full service restaurants were: Applebee’s, Olive Garden, Chili’s, Red Lobster, IHOP, Denny’s, Outback Steakhouse, Buffalo Wild Wings, Cracker Barrel, and TGI Friday’s. FV = non-fried fruit or vegetable.

## Results

### Child demographics and frequency of eating at restaurants

The total weighted sample consisted of 43.8% tweens (aged 8–12, n = 516) and 56.2% teens (aged 13–18, n = 662) and 47.8% boys (n = 563) and 52.2% (n = 615) girls. The racial/ethnic distribution was: 58% White, 19% Hispanic, 14% Black/African-American, 3% Asian/Pacific Islander, 1% Native American/Alaskan Native, and 3% mixed race, with the remaining 2% indicating another race or declining to answer. Nearly all children in the sample reported eating food from a restaurant at least a few times per year (99%), with 55.8% eating at restaurants at least a few times per month and 13.6% eating at restaurants at least once per week. Sixty-seven percent of children reported eating take-out from restaurants at least a few times per month, and 19.4% reported eating take-out weekly. When asked what they do when they eat at a restaurant and there is too much food, the majority of children reported taking it home (69%); the next most common answers were leaving the food on their plate (12%) and trying to finish it anyway (9%). Only 3% of children selected a statement indicating that there was never too much food. Fifty-nine percent of children reported ordering kids’ meals. The likelihood of ordering kids' meals differed by age group (*B* = -1.21; 95% CI: -1.31, -1.10; *p* = .000), with 84.0% of tweens and 40.2% of teens reporting that they ever order kids' meals.

### Children’s attitudes about fruit, vegetable, and French Fry side dishes in kids' meals

Of the children who reported ordering kids' meals, more than half said that they would be somewhat or very likely to order a kid's meal that came with vegetables (56.2% of tweens; 54.8% of teens) or fruits (78.9% of tweens; 73.0% of teens). Most children also reported being somewhat or very likely to order a kid's meal that came with French fries (89.8% of tweens and 86.7% of teens). When asked how they would feel if their kid's meal came with a vegetable or fruit but not French fries, 32.8% of children reported that they would be somewhat or very unhappy (34.1% of tweens and 30.7% of teens), 34.9% would be neither happy nor unhappy (32.9% of tweens and 38.1% of teens), and 32.3% of children would be somewhat or very happy (33.0% of tweens and 31.2% of teens). While two-thirds of children reported non-negative attitudes about this substitution, there were individual differences in children’s attitudes (Table 3). Overall, children who ate at restaurants less frequently, children who ate take-out from restaurants more frequently, and boys were less accepting of fruit and vegetable side dishes and were more likely to endorse French fries as side dishes. Compared to teens, tweens were more likely to endorse French fries but were also more likely to accept fruits.

**Table 3:** Frequency of eating food at and from restaurants as predictors of children’s attitudes about vegetable, fruit, and French fry side dishes

**Model**	***B***	**95% CI**	***p-value***
**Predictor: Frequency of eating food at restaurants**			
*How likely to order vegetable side dish (higher = more likely)*			
**Frequency (higher = more often)**	**.14**	**.05, .24**	**.004**
Age group (1 = teens)	-.03	-.19, .13	.694
**Sex (1 = female)**	**.25**	**.09, .40**	**.002**
*How likely to order French fry side dish (higher = more likely)*			
Frequency (higher = more often)	-.06	-.13, .01	.109
**Age group (1 = teens)**	**-.12**	**-.24,** -**.00**	**.044**
**Sex (1 = female)**	**-.21**	**-.33,** -**.09**	**.000**
*How likely to order fruit side dish (higher = more likely)*			
Frequency (higher = more often)	.03	-.06, .12	.543
**Age group (1 = teens)**	**-.21**	**-.36,** -**.07**	**.004**
**Sex (1 = female)**	**.17**	**.03, .32**	**.017**
*How happy if side dish was fruit/vegetable and not French fries (higher = happier)*			
**Frequency (higher = more often)**	**.14**	**.03, .25**	**.017**
Age group (1 = teens)	-.01	-.19, .18	.955
**Sex (1 = female)**	**.29**	**.12, .47**	**.001**
**Predictor: Frequency of eating food from restaurants (take-out)**			
*How likely to order vegetable side dish (higher = more likely)*			
Frequency (higher = more often)	-.06	-.15, .04	.218
Age group (1 = teens)	-.06	-.22, .10	.438
**Sex (1 = female)**	**.23**	**.08, .39**	**.003**
*How likely to order French fry side dish (higher = more likely)*			
**Frequency (higher = more often)**	**.15**	**.08, .22**	**.000**
Age group (1 = teens)	-.10	-.22, .02	.087
**Sex (1 = female)**	**-.19**	**-.31,** -**.08**	**.001**
*How likely to order fruit side dish (higher = more likely)*			
Frequency (higher = more often)	-.01	-.09, .08	.885
**Age group (1 = teens)**	**-.22**	**-.37,** -**.07**	**.003**
**Sex (1 = female)**	**.17**	**.03, .31**	**.019**
*How happy if side dish was fruit/vegetable and not French fries (higher = happier)*			
**Frequency (higher = more often)**	**-.16**	**-.26,** -**.05**	**.006**
Age group (1 = teens)	-.04	-.22, .14	.665
**Sex (1 = female)**	**.27**	**.09, .45**	**.003**

Note: Bolded rows indicate statistically significant results (*p* < .05).

**Figure 1:**
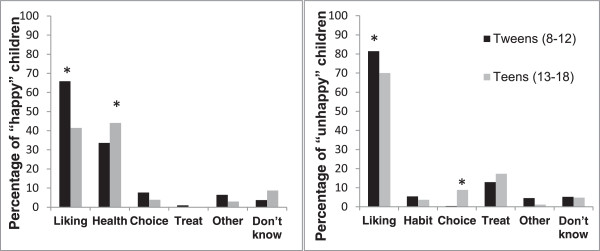
**Categorization of children’s open-ended responses explaining why they would be happy or unhappy if their kid’s meal came with a fruit/vegetable instead of French fries.** One-third of children reported that they would be happy or somewhat happy, and these children are included in the left panel. One-third of children reported that they would be unhappy or somewhat unhappy, and these children are included in the right panel. For the children who would be happy if their kid’s meal came with a fruit/vegetable instead of French fries, the most common reasons were related to liking/taste (of fruits/vegetables; 57.1%) and health (41.9%). For the children who would be unhappy if their kid’s meal came with a fruit/vegetable instead of French fries, liking/taste (of French fries) was the most common reason (77.4%). Children's coded, open-ended responses could fall into more than one category, and category descriptions are in Table 1. Note: * = significant age group difference at *p* < .05.

As shown in Figure 1, children's most common reasons for being happy about kids’ meals coming with a vegetable or fruit instead of French fries were "liking/taste" (57.1%) and "health" (41.9%). In this case, liking-related responses referred to liking or preferring fruits or vegetables or disliking French fries. Tweens were 2.9 times more likely to endorse liking/taste as a reason, compared to teens (66.3% of tweens vs. 41.5% of teens; 95% CI: 1.55, 5.24; *p* = .001), and teens were 2.2 times more likely to endorse health as a reason, compared to tweens (33.6% of tweens vs. 55.9% of teens; 95% CI: 1.19, 3.94; *p =* .012). Among these children, neither sex nor frequency of eating at restaurants predicted the likelihood of endorsing liking/taste or health, and neither sex, age group, nor frequency of eating at restaurants predicted the likelihood of endorsing any of the other categories.

Children's most common reason for being unhappy if their kid’s meal came with a vegetable or fruit instead of French fries was liking/taste (77.4%). In this case, liking-related responses referred to liking or preferring French fries or disliking fruits or vegetables. Tweens were 2.1 times more likely to endorse liking/taste as a reason, compared to teens (81.5% of tweens vs. 70.0% of teens; 95% CI: 1.04, 4.10; *p* = .038). Neither sex nor frequency of eating at restaurants predicted the likelihood of endorsing liking/taste. Among these children, significant predictors of endorsing other, less common categories were as follows: teens were more likely to endorse "wanting choices" as a reason for being unhappy (OR: 19.58; 95% CI: 1.47, 260.3; *p* = .024), and those eating at restaurants less frequently were more likely to give a response categorized as “other” (OR: 9.27; 95% CI: 2.22, 38.69; *p* = .002). For the most part, these results were consistent when substituting the frequency of eating take-out from restaurants into the models (as opposed to the frequency of eating at restaurants), with two exceptions: children eating take-out less often were more likely to endorse health as a reason for being happy (OR: 1.47; 95% CI: 1.01, 2.14; *p* = .046), and children eating take-out less often were more likely to endorse “other” reasons for being unhappy (OR: 3.12; 95% CI: 1.02, 9.55; *p* = .046).

### Current availability of fruits and vegetables as side dishes on children’s menus

**Figure 2:**
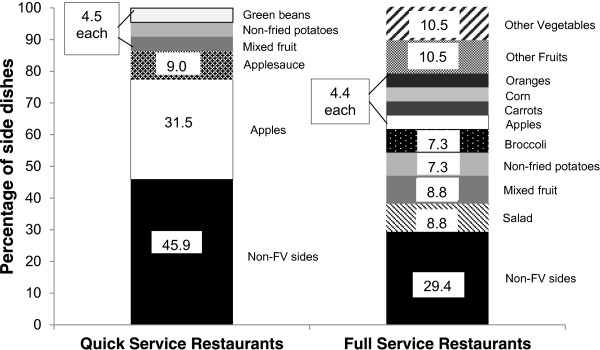
**Prevalence of side dish offerings on children’s menus at quick service (QSR) and full service restaurants (FSR).** The prevalence of fruit and vegetable (FV) and non-FV side dishes across children’s menus at the top 10 QSR and top 10 FSR are shown. FV sides consist of all non-fried FV and include legumes. Non-FV sides include all other sides, such as French fries, rice, and biscuits. The solid black blocks represent the prevalence of non-FV side dish options. Together, the remaining blocks show the overall prevalence of FV side dishes, and they are divided to further illustrate the specific FV side dishes available. The most common FV side dish items are depicted individually, and less common items are collapsed into “Other Fruits” (applesauce, grapes, pineapple, berries) and “Other Vegetables” (green beans, celery, mixed vegetables, cole slaw, other beans, greens). Full service restaurants had more variability among their FV side dish items, compared to QSR.

Nearly all of the children’s menus analyzed had at least one FV side dish available (95% overall; 90% of QSR and 100% of FSR). While restaurants were consistent in terms of FV availability, there was variability in the prevalence of FV side dish items across restaurants. On average, children’s menus at QSR offered 2.3 side dish options (SD = 1.1), and on average, 54.2% of side dish options were a fruit or vegetable (range = 0 to 3 FV items). On average, children’s menus at FSR offered 7.9 side dish options (SD = 6.5), and on average, 70.1% of these were a fruit or vegetable (range = 2 to 12 FV items). There was also variability in the extent to which FV items were served as the default, or automatic, side dish. Forty percent of QSR provided kids’ meals that always included a FV as the default side dish, and 60% never included FV as the default side dish. Twenty percent of FSR provided kids’ meals that always included a FV as the default side dish, while 10% included FV as the default side for some but not all main dishes, and 70% never included FV as default side dishes. With regards to the specific FV side dishes offered, there were 19 different FV side dish items offered across children's menus at the 20 restaurants. The most commonly available FV side dishes, and corresponding frequencies, were: apples (10), mixed fruit (7), non-fried potatoes (6), salad (6), broccoli (5), and applesauce (4). As shown in Figure 2, there was more variability in side dish offerings at FSR compared to QSR. In particular, vegetables were less available in QSR, with only one QSR offering any vegetable options (mashed potatoes and green beans).

Among the 11 restaurants eligible for the analysis of energy content, a serving of French fries had as few as 100 calories and as many as 440 (M = 277 kcal; SD = 105). Of the common FV sides, mashed potatoes had the highest energy content (M = 135 kcal; SD = 7), and apple slices and broccoli without dips or dressings had the lowest energy content (15 and 25 calories respectively). The substitution of a common FV item for French fries consistently led to an average reduction of at least 170 calories (Table 4). The highest energy reductions were achieved by substituting applesauce or broccoli in place of French fries.

**Table 4:** Reductions in kid's meal energy content when substituting FV side dishes for French fries, in 11 restaurants offering French fries and at least 1 common FV side dish

	**N**	**Mean calories ± SD**	**Range**	**Calories saved vs. fries ± SD**	**Range of calorie savings**
Fries^a^	11	277 ± 105	100-440	n/a	n/a
Apple slices^b^	6	68 ± 48	15-140	171 ± 74	85-290
Apple sauce	2	70 ± 28	40-90	300 ± 71	250-350
Broccoli	3	58 ± 45	25-109	283 ± 128	160-415
Mashed potatoes^b^	2	135 ± 7	130-140	180 ± 156	70-290
Salad^b^	3	65 ± 39	40-110	242 ± 148	90-385

^a^Includes French fries, waffle fries, curly fries, and tater tots.

^b^Includes dipping sauce, gravy, or dressing when indicated in nutrition facts (n = 6).

Note: Substitutions were calculated within restaurants and then averaged across the total sample of restaurants for that FV side dish item. QSR and FSR are included. The analysis excludes baked cinnamon apple slices which, at 270 calories, were an outlier. When included, the overall mean calories for apples increase to 97 ± 88, and mean calories saved via substitution decrease to 136 ± 113, and the range expands to -70-290. The analysis includes one restaurant in which French fries and apples are served together by default, illustrating the energy reduction that could be achieved if only apples were offered. If this restaurant were excluded, the overall mean calories for apples increase to 79 ± 45, mean calories saved via substitution increase to 188 ± 68, and the range narrows to 110–290.

## Discussion

Results confirm that American children are consuming food from restaurants frequently and provide new evidence that the majority report non-negative attitudes about receiving FV side dishes with kids' meals. An analysis of online menus from leading QSR and FSR showed that nearly all were offering FV side dishes in April 2013. Yet these options were typically offered alongside energy-dense options, like French fries, and only a minority of restaurants offered a FV item as the default side dish. These results reflect recent efforts to improve children’s menus but also illustrate that more can be done to shift norms around "children's food" and encourage healthy eating in restaurant contexts.

While the current results suggest that many children would accept FV side dishes, they also highlight that tailored strategies may be warranted to promote FV acceptance in certain subgroups: namely, boys, children eating at restaurants less frequently, and children eating take-out more frequently. Findings that these subgroups were less likely to endorse FV side dishes could be used to shape strategies to encourage increased acceptance of these foods, such as incorporating children from these demographics into advertisements and public service announcements promoting FV. In some ways, these subgroup differences are good news: for example, the relationship between a *higher* frequency of eating at restaurants and increased likelihood of accepting FV side dishes is promising in terms of the potential public health impact of adding more healthy options. It is important to acknowledge that results were in the opposite direction when investigating the frequency of eating take-out; more research is needed to explore relationships between these two modalities of consuming restaurant-prepared food, demographics, food preferences, and intake. Eating food from restaurants may be perceived as a special occasion [36,37], so nutrition may not be a main concern of families in these contexts; yet, this view may not be warranted when children consume restaurant foods frequently. The results linking higher frequency of eating take-out with a lower likelihood of selecting healthy options highlights the possibility that a reframing of our view of take-out foods in particular may be warranted. Findings also showed that, while younger children were more likely to accept French fries, they were also more likely to accept fruit, possibly demonstrating a cohort effect where younger children have become accustomed to recent menu changes incorporating fruits as side dishes.

The open-ended portion of the current results provides additional insights into children’s attitudes about substituting FV side dishes in place of French fries. Consistent with past research, taste emerged as a key factor influencing children's food preferences [32,33,38]; this was true for both children who would be happy and those who would be unhappy about the substitution. For the former group, this meant that children would be happy because they like or prefer vegetables or fruits, and for the latter, this meant that children would be unhappy because they like or prefer French fries. While liking/taste was a popular reason overall, younger children were more likely than teens to discuss liking or taste in their open-ended response. Together, these findings suggest that emphasizing flavor may be an effective means of increasing children’s consumption of FV side dishes, particularly among younger children. Besides liking/taste, another factor that emerged as a common reason that children would be happy about FV side dishes was health, and this reason was more common among teens than tweens. Yet we argue that taste remains the most important factor, given its prevalence among both the happy and unhappy children, its increased importance in the younger children who were more likely to order kids' meals, and evidence showing that short-term rewards like taste often outweigh long-term rewards like health in decision making, particularly in those who find palatable foods to be highly reinforcing [39]. Still, it is encouraging to find that health concerns resonate with some children in the context of the childhood obesity epidemic.

Children's perceptions about healthier side dish options can inform action during a time in which it is important to encourage improvements to children's dietary intake in restaurant settings. Our analysis of menus at leading QSR and FSR showed that there has been some progress in this area since earlier assessments [3]; for example, FV side dishes were available at nearly all restaurants in April 2013. However, these were the default side dish at the minority of restaurants in both restaurant segments. Fruits or vegetables were more likely to be default side dishes at QSR than FSR, but there was a smaller variety of FV offerings at QSR, with a notable dearth of vegetable offerings, such that only one QSR studied offered any non-fried vegetable side dishes. Additionally, even though the majority of children expressed non-negative attitudes about FV side dishes, more than 85% of children reported that they would be likely to order French fries as a side dish, suggesting that promoting healthier side dishes necessitates strategies that go beyond simply offering a FV option in direct competition with palatable, energy-dense alternatives. Given children’s focus on taste and their past experiences with these energy-dense options, simply making FV sides available is unlikely to maximize the ordering and consumption of these options.

Instead, one potential way to promote the consumption of FV side dishes is repeatedly exposing children to FV when they order food from restaurants, by making FV default side dishes that automatically come with their meal. This strategy may be particularly important for younger children, who were more likely to order from children's menus, were more likely to endorse French fries, and for whom there is evidence that taste preferences are modifiable through strategies like repeated exposure [40]. Increased adoption of this strategy could shift perceived norms around restaurant meals and children's food. It could also be particularly important for increasing acceptance and consumption of vegetables, given that children's intake of vegetables is further from dietary recommendations compared to fruits [41], and given the dearth of default vegetable offerings on children's menus.

Our analysis of nutrition information shows that FV defaults have the potential to lower the energy density of children's meals. Substituting commonly offered FV side dishes for French fries could achieve average reductions of 171 to 300 calories per meal. This change, aggregated over repeat visits, could have a measurable impact on energy balance. We also noted variability in energy content across FV items and within the same type of item across restaurants. For example, the energy content of apple slices depended on portion size and whether they were served alone, with a dipping sauce, or baked with other ingredients (range: 15–270 calories). Similarly, given different portion sizes across establishments, the energy in a child’s serving of French fries could quadruple from one restaurant to the next, ranging from 100 to 440 calories, the latter of which contributes the amount of energy appropriate for an entire meal for a sedentary 8-year-old child [9]. These estimations suggest that incorporating healthier side dish options, particularly those of a moderate portion size that are not prepared with added energy-dense ingredients, and encouraging children to consume them, by making them the only option, the default option, or the most attractive option, could have a measurable impact on intake.

In estimating the potential energy reductions afforded by FV default sides, there are two important caveats to note. First, previous research has demonstrated that energy content information from restaurants is accurate on average, but that there are discrepancies between stated and actual energy content on some types of menu items. It has been shown that, in FSRs, energy content was understated for higher-energy items and overstated for lower-energy items [42]. Thus, for some restaurants in our sample, it is possible that the estimated energy reductions when substituting FV for French fries may be overstated. Additionally, it is important to note that substituting FV for French fries will result in an actual, net decrease in energy intake only if patrons do not compensate for the energy reductions through other meal components. The actual impact of such substitutions on children's consumption has yet to be determined, and current evidence supports competing hypotheses. On one hand, experimental research on energy density suggests that children consume a consistent weight of food, and thus, it follows that substituting less energy-dense foods for more energy-dense foods should decrease total energy intake [43,44]. On the other hand, it could be argued that substituting FV for French fries is a more salient shift than those represented by the conditions in energy density experiments, and perceiving side dishes as healthy may lead to overeating [45]: for example, the child and/or parent may choose to order an energy-dense dessert or sugar-sweetened beverage to accompany a "healthier" kid's meal. Future research should use objective methods to explore the extent to which the incorporation of FV side dishes impacts the energy consumed by children within a meal and throughout the day.

Limitations of the current study include potential social desirability in children’s reports, which could result in a discrepancy between what was reported and children’s actual behaviors and preferences. Lower reported frequencies of eating food from restaurants, compared to other national data [2], suggest that this may have occurred, although it is also possible that these differences emerged for reasons besides social desirability. While the use of sampling weights make results generalizable to US children ages 8 to 18 years based on demographics, it is possible that representativeness in terms of restaurant patronage was not achieved, which would affect the generalizability of the current results. Alternatively, it is possible that the discrepancy is due to methodological differences or historical trends. It is not possible to parse these possibilities apart in the current study. Another limitation is the lack of a priori measurement work on the survey, although results provide initial evidence of reliability and validity of survey questions. Finally, publically available menus and nutrition information may not fully reflect children's experiences in restaurant settings. As mentioned, stated energy content does not always match actual energy content [42]. Further, the depiction of side dishes on online menus may not reflect the meal pairings that are most salient to children via advertisements or sensory experiences in restaurants. Additional research using a mixture of methodologies can contribute to a comprehensive picture of children's current experiences in restaurants, including how their attitudes and experiences translate into behaviors and consumption patterns.

Making FV side dishes more prevalent and automatic and evaluating the impact of such changes could increase the momentum following initial, positive changes that restaurants have made in recent years [5]. There is evidence to suggest that a focus on healthier children's meals has the potential to be well-received and economically sustainable [46], highlighting the potential value of such shifts from the restaurant perspective. While the focus of this study was on side dishes, this is only one aspect of the energy consumed in restaurants and only one part of the overall secular trend toward healthier menus for children. In order to accelerate progress in this area, a comprehensive consideration of all aspects of restaurant meals is warranted. Other meal components that could contribute excess energy include, but are not limited to, sugar-sweetened beverages, desserts, and main dishes [29]. Additionally, while some older children reported ordering kids' meals, this was more likely in the younger age group. This finding suggests that efforts to impact energy intake in restaurants across child age groups necessitates a consideration of the overall menu, which could have a positive impact on the family more broadly. The age group differences found in this study also suggest that subsequent research may benefit from a more in-depth consideration of issues impacting younger vs. older children, including their engagement with "adult" menu items, the role of price and financial independence, and developmental changes in attitudes about restaurant foods.

## Conclusions

Overall, the current results suggest that the promotion of FV side dishes show promise as part of comprehensive efforts to improve children's dietary intake in restaurant settings. In recent years, there has been increased demand for healthier options in restaurants, and restaurants have made some progress in this area, with some success. Fruit and vegetable side dish options are now available at most restaurants frequented by children, but they are seldom the default and typically compete with more energy-dense options. There is room for continued improvement, in terms of promoting and normalizing the consumption of healthier side dishes and in terms of menus more broadly. Promising strategies include substituting or displacing energy-dense default side dishes with FV, as part of comprehensive efforts to increase the healthfulness of meals consumed by children in restaurant settings. These strategies have the potential to reduce energy imbalance. Given the high rates at which children consume food away from home, it is important to continue to test and promote these and other strategies, in order to make healthy choices easier for children and families in restaurant settings.

## Abbreviations

FV: Fruits and vegetables; QSR: Quick service restaurants; FSR: Full service restaurants; U.S.: United States.

## Competing interests

The authors have no competing interests.

## Authors’ contributions

SAF, FD, and SS contributed to study design, data analysis, data interpretation, writing, and revising; PD and MEN contributed to study design and revising; and KW and CDE contributed to study design, data interpretation, writing, and revising. All authors read and approved the final manuscript.
